# A Will

**Published:** 1879-07

**Authors:** 


### A WILL.

The defective preparation and execution of wills is not always explainable by the statement that the maker was ignorant or careless.

Josiah Bacon, who was recently murdered in San Francisco, left a will which has been refused probate in Massachusetts because it has not the legal number of witnesses, three being required, and this instrument having only two. Mr. Bacon was a regularly educated lawyer, who was doubtless familiar with the forms and requirements for such instruments. He wrote the will and attended to its execution. The old saw about the man who acts as his own lawyer has in this case additional confirmation.

A great many lawyers' wills, drawn by themselves, have been set aside. The explanation usually given is rather creditable to them—which is that they do not take as much pains for themselves as they do for their clients.

